# The Management of Older Adults with Pancreatic Adenocarcinoma

**DOI:** 10.3390/geriatrics3040085

**Published:** 2018-11-26

**Authors:** John R. Ogden, Hao Xie, Wen Wee Ma, Joleen M. Hubbard

**Affiliations:** 1 Division of Internal Medicine, Department of Medicine, Mayo Clinic, Rochester, MN 55905 USA; Ogden.John@mayo.edu; 2 Division of Medical Oncology, Department of Oncology, Mayo Clinic, Rochester, MN 55905, USA; Xie.Hao@mayo.edu (H.X.); ma.wen@mayo.edu (W.W.M.)

**Keywords:** pancreatic adenocarcinoma, older adults, disease management, localized disease, metastatic disease

## Abstract

Pancreatic cancer is the eleventh most common cancer, yet it is the third leading cause of mortality. It is also largely a disease of older adults, with the median age of 71 at diagnosis in the US, with <1% of diagnoses occurring prior to age 50. Current NCCN guidelines recommend surgery for localized disease, followed by adjuvant therapy and/or consideration of enrollment in a clinical trial. For metastatic disease, current guidelines recommend clinical trial enrollment or systemic chemotherapy based on results from the landmark ACCORD-11 and MPACT trials. However, these trials focused heavily on younger, more fit patients, with the ACCORD-11 trial excluding patients over age 75 and the MPACT trial having 92% of its patients with a Karnofsky performance score >80. This article summarizes the available evidence in current literature in regards to the best treatment options for older adults, who represent the majority of pancreatic cancer diagnoses.

## 1. Introduction

Pancreatic cancer has an expected incidence of 55,440 new cases and 44,330 deaths in 2018 in the United States alone [1]. Although it is the eleventh most common cancer, it is the third leading cause of cancer-related death [2]. It is also largely a disease of older adults, with the median age of 70 at diagnosis in the US, with 11% of diagnoses occurring at age 54 or younger, according to the SEER database [3]. Pancreatic cancer is typically divided into three categories: localized, locally advanced, and metastatic. Currently, localized disease offers the only chance for cure. Unfortunately, only about 10% of diagnoses are made at the localized stage, largely because symptoms are nonspecific until advanced stage. Because of this, five-year overall survival for pancreatic cancer remains dismally low at 8% [1].

For localized disease, the 2017 National Comprehensive Cancer Network (NCCN) guideline recommends surgery for patients with good performance status and consideration of enrollment in a clinical trial during their treatment. Adjuvant therapy consisting of chemotherapy and/or concurrent chemo-radiation should be considered following resection [4]. If a patient received neoadjuvant treatment prior to surgical resection, additional adjuvant chemotherapy should still be considered. For metastatic disease, NCCN guideline recommends preferably clinical trial enrollment, FOLFIRINOX therapy, or gemcitabine-based therapy, typically in combination with nab-paclitaxel. The efficacy of FOLFIRINOX regimen was shown to be superior to Gemcitabine alone in the ACCORD-11 trial [5], and the combination of Gemcitabine plus nab-paclitaxel was shown to be superior to Gemcitabine alone in the MPACT trial [6]. However, these trials focused heavily on younger, more fit patients, with the ACCORD-11 trial excluding patients over age 75 and the MPACT trial having 92% of its patients with a Karnofsky performance score >80.

This article summarizes the available evidence in current literature in regards to the best treatment options for older adults, who represent the majority of pancreatic cancer diagnoses. We did an extensive search of PubMed using the terms “localized pancreatic cancer”, “locally advanced pancreatic cancer”, “metastatic pancreatic cancer”, “treatment”, and “elderly” to find data on what the best treatment options might be for older patients and what discrepancies, if any, exist in the current literature. Table 1 summarizes the articles that were selected for review of the management of localized disease.

## 2. Localized Disease

Localized (or resectable) disease is defined as disease that is confined to the pancreas without extensive invasion of local structures [4]. Specifically, no arterial tumor contact with the celiac, superior mesenteric, or common hepatic arteries, as well as no venous contact with the superior mesenteric vein or ≤180 degree contact with the portal vein without vein contour irregularity. It is optimally treated with surgery, chemotherapy, with/without concurrent chemo-radiation therapy. 

### 2.1. Who among the Elderly Received Treatment?

In our review of the literature, we found that elderly patients were significantly less likely to undergo surgical resection compared to their younger counterparts, irrespective of comorbidity status. In a retrospective analysis of the Surveillance, Epidemiology, and End Results (SEER) program database on a total of 2229 patients age 65 and older, the authors found that, in practice, increasing age was an independent variable for foregoing surgical resection. Compared with patients age 65–69, the odds ratio (OR) for receiving surgical resection for patients age 70–74 was 0.84 (95% CI 0.62–1.14), age 75–79 was 0.74 (0.54–1.01), age 80–84 was 0.52 (0.37–0.72) and age ≥85 was 0.39 (0.24–0.62). Other factors that favored receiving surgery were male gender, with females having an OR of 0.85 (0.69–1.05); white race, with African Americans having an OR of 0.99 (0.65–1.52); and other races, with an OR of 0.73 (0.52–1.02); however these factors were not statistically significant. Tumor size was also a significant factor in decision for surgery, with tumors ≥2 cm having an OR of 0.33 (0.21–0.52) compared with smaller tumors [7]. In another study by Riall et al., age again was found to be a significant predictor of who received evaluation for surgery and surgery, regardless of comorbidity status, with resection rates of 39% in those <70 years of age, whereas 5% of patients aged ≥85 underwent resection [8].

### 2.2. What Are the Risk/Benefit Considerations for Elderly Patients Undergoing Surgical Resection?

Previous studies demonstrated that elderly patients who underwent surgery did much better in terms of overall survival compared with patients who did not undergo surgery. A retrospective study by Riall et al. identified 9553 patients age ≥65 from the SEER database diagnosed with locoregional pancreatic adenocarcinoma between 1992 and 2005. Of these, 69% were evaluated by a surgeon and 25% underwent surgical resection. Here, surgical resection was shown to have significant improvement in overall survival, with similar survival benefits regardless of age group. Compared to the unresected patients, the HR for survival for patients <70 who underwent surgery was 0.46 (0.41–0.53) compared with unresected patients, a HR of 0.51 (0.46–0.58) for age 70–74, 0.47 (0.41–0.53) for age 75–79, 0.43 (0.37–0.50) for age 80–84, and a HR of 0.35 (0.26–0.48) for age ≥85. While the survival advantage among resected patients was seen across all age groups, the study did find increased 30 day mortality in older age groups, with in hospital mortality being 7% in patients aged 65–69 versus 11.5% in those aged 85 and older, although not statistically significant (*p* = 0.41) [8].

The results of this retrospective study agreed with other similar studies [7,9,10,11,12,13,14,15], including a study by van der Geest et al. of 3845 patients from the Netherlands Cancer Registry who underwent resection for primary pancreatic or periampullary carcinoma. Here, they found that octogenarians had greater 30 day and 90 day postoperative mortality compared to younger patients, with an odds ratio (OR) of 2.26 (1.25–4.06) for 30 day mortality and an OR of 2.48 (1.53–4.02) for 90 day mortality compared with patients age ≤70. The survival among octogenarians who underwent surgery were similar to other age groups at one (53%), three (21%), and five years (13%) [9].

### 2.3. Which Is a Better Treatment for Localized Disease in the Elderly: Surgery or Chemotherapy?

Comparing surgery alone versus chemotherapy alone, the previously mentioned study by Marmor et al. evaluated SEER data among 2229 patients ≥65. This study found a significant unadjusted Kaplan Meier mortality benefit of a median OS within the surgery group compared to the chemotherapy only group (15 months versus 10 months *p* < 0.001), however this benefit did attenuate with age (13 months versus 10 months in those age 80 or older *p* < 0.01) [7]. 

### 2.4. What Is the Role of Adjuvant Chemotherapy?

The combination of surgery and adjuvant chemotherapy has better outcomes in terms of survival compared with surgery alone. The ESPAC-1 was the first trial to demonstrate a survival advantage with adjuvant chemotherapy in 2004. This phase III, randomized trial analyzed patients who had undergone resection for pancreatic ductal adenocarcinoma and randomized them into 4 separate groups: treatment with chemotherapy alone (fluorouracil), chemoradiotherapy alone (20 Gy over a two-week period plus concurrent fluorouracil), chemoradiotherapy followed by chemotherapy, and observation. When comparing adjuvant chemotherapy with observation, the median survival in patients receiving adjuvant chemotherapy was 20.1 months, compared with 15.5 months in those who did not, with a hazard ratio of 0.71 (95% CI 0.55–0.92). Additionally, questionnaires regarding quality of life showed no significant differences between those receiving chemotherapy and those who did not [16]. The study included patients age 34–82, and while 62% of patients were age ≥65, the overall survival benefits were not stratified by age in this study. 

The CONKO-001 trial later demonstrated that adjuvant gemcitabine improved survival following surgery compared with no adjuvant chemotherapy. In this trial, a total of 368 patients were randomized into a treatment group with surgery plus adjuvant gemcitabine, and a control group with surgery alone in an intention to treat analysis. This trial showed that adjuvant treatment with gemcitabine for 6 months following surgery led to statistically significant improvements compared to surgery alone in terms of disease free survival (13.4 months versus 6.7 months), 5-year survival (20.7% versus 10.4%) and overall survival (22.8 months versus 20.2 months). This benefit was not lost with age, as multivariate analysis of age ≥65 showed a hazard ratio of 1.24 (0.99–1.56) [17].

Comparing gemcitabine and 5-FU, the ESPAC-3 trial showed that median overall survival was similar between the two treatment groups, with median survival of 23.6 months and 23.0 months, respectively (*p* = 0.39), with median progression free survival of 14.3 months versus 14.1 months. The 5-FU group was noted to have significantly more grade 3/4 stomatitis and diarrhea (*p* < 0.001), whereas the Gemcitabine group was noted to have increased grade 3/4 hematologic toxicity (*p* = 0.003). Quality-of-life (QOL) domain scores were calculated according to the EORTC QLQ-30 scoring manual, which assesses multiple categories such as pain, fatigue, nausea, and vomiting, emotional and physical functioning as well as cognitive and social functioning. These QOL scores showed no significant difference in overall quality of life between the two groups [18]. 

Compounding upon the CONKO-001 trial, the ESPAC-4 trial showed the benefits of adjuvant chemotherapy when comparing gemcitabine plus capecitabine versus gemcitabine alone. In the ESPAC-4 trial, 760 patients were divided into the two treatment groups in an intention to treat analysis. The ESPAC-4 trial showed an increase in overall survival of 28 months in the combination arm versus 25.5 months in the Gemcitabine alone control arm. This came at the expense of more grade 3 or 4 toxicities in the combination group, with a total of 608 grade 3–4 adverse events reported by 63% of patients in the combination group, compared with 481 grade 3–4 adverse events in 54% of patients in the monotherapy group. The benefit of combination treatment remained the same regardless of age, with a Forest plot showing a HR of 0.82 in patients age < 65 (n = 382), and a HR of 0.81 in patients age ≥65 (n = 348) [19].

More recently, the PRODIGE-24 trial compared FOLFIRINOX and gemcitabine in the adjuvant setting, given the superiority of FOLFIRINOX that has been demonstrated in the metastatic setting. This phase III, multicenter trial randomly assigned 493 patients into the two treatment groups. With a median follow up time of 30.5 months, the median OS was found to be significantly longer in the FOLFIRINOX group at 54.4 months versus 34.8 months in the gemcitabine arm (HR = 0.59; 95% CI 0.46–0.76). This trial included patients aged 18-79 with WHO PS ≤ 1. However, we are awaiting for further analysis that would be necessary to show if this benefit holds true for the older adult patient population [20].

### 2.5. Is There Any Role for Radiation Therapy in Localized Disease?

In the previously mentioned ESPAC-1 trial, when they compared patients who received chemoradiation with those who did not they found that chemoradiation resulted in worse outcomes, with a HR for death of 1.28 (95% CI 0.99–1.66, *p* = 0.05) [16]. This data, however, comes from the 2004 ESPAC-1 trial, and therefore may not be applicable to today’s practice given the advancements that have occurred in the field of radiation oncology. Highlighting the unintended consequence ESPAC-1 had on radiation therapy, a study conducted by Shinohara et al. analyzed the SEER database from the 5-year time period preceding and following publication of ESPAC-1 to see how it influenced use of postoperative radiation therapy (PORT). There were 1,628 patients in the pre-publication period, compared to 2194 patients in the post-publication period. Their study found that PORT was used significantly less in the post-publication period, with the odds ratio for PORT in the pre-publication period of 1.19 (95% CI 1.04–1.35) [21]. 

However, since then there have been some small studies showing benefit to the addition of radiation to chemotherapy. The first randomized study showing this was reported by the Gastrointestinal Tumor Study Group, noting that 22 patients who underwent observation had a median survival of 11 months, compared with a median survival of 20 months in 21 patients who underwent chemoradiation therapy (*p* = 0.035) [22]. 

Perhaps the most significant trial to assess the role of chemoradiation therapy was the LAP-07 trial, which was a phase III randomized trial comparing the role of chemoradiotherapy versus chemotherapy alone in the postoperative setting. Unfortunately, this trial was conducted primarily in ‘younger’ and ‘fitter’ patients and excluded those over the age of 70, and the average patient age was 63. In this study, 449 patients were enrolled and received gemcitabine or gemcitabine plus capecitabine after surgery. They then underwent a second randomization involving patients with progression-free disease after four months. In this second randomization, 136 patients received 2 additional months of the same chemotherapy, and 133 underwent chemoradiotherapy. This study found no significant difference in median overall survival between the two groups, with the chemotherapy group having a median OS of 16.5 months and the chemoradiotherapy group having a median OS of 15.2 months HR 1.03; CI 0.79–1.34) [23]. 

A recent systematic review conducted by Ciabatti et al. analyzed 11 publications that included 1830 patients aged 65 and older looking at the safety of radiation therapy with and without chemotherapy in the treatment of unresectable pancreatic adenocarcinoma. They concluded that radiation therapy in the elderly population was a viable and safe option, with grade ≥3 (0–52.6%, median 0.5%) and ≥2 (0–15%, median 0%) toxicities similar to that of the general pancreatic adenocarcinoma population, making this a reasonable choice in older adults in whom radiation is being considered [24].

The recently published results of the phase III PREOPANC-1 trial support the use of neoadjuvant chemoradiotherapy in borderline resectable disease. This trial randomized 246 patients with borderline resectable pancreatic cancer to receive either immediate surgery (127 patients) or preoperative chemoradiotherapy (119) in an intention to treat analysis. The early results are quite promising, with the chemoradiotherapy arm having a significantly improved OS compared with immediate surgery (17.1 months versus 13.5 months; HR 0.71; *p* = 0.047). The study also demonstrates improved margin free resection rates (65% versus 31%, *p* < 0.001) in the neoadjuvant group. There was no significant difference in observed grade ≥3 toxicities in either group (*p* = 0.17). While this abstract does show a clear benefit to neoadjuvant chemoradiotherapy in borderline resectable disease, the abstract does not clarify patient age and thus may not be applicable to older adults [25].

While overall there is little to no data or evidence to support or contradict the use of radiation therapy in addition to chemotherapy, advances in radiation therapy could show to provide benefit in the treatment of pancreatic cancer in the proper setting, and larger studies are needed to truly assess its potential role in treatment.

## 3. Metastatic Disease

### 3.1. What Is the Current Standard?

Metastatic pancreatic adenocarcinoma is not amenable to surgical resection, therefore systemic chemotherapy is the standard of care in attempting to control metastatic disease. The trial by Burris et al. in 1997 was essential in establishing a new standard when it showed that Gemcitabine was superior to 5-FU in advanced disease [30].

Another phase III trial published in 2007 by Moore et al. showed that the addition of Erlotinib to Gemcitabine improved outcomes. This trial randomized 569 patients into the two treatment groups and showed a statistically but not clinically significant effect on survival, with a median OS of 6.24 months versus 5.91 months, and a HR of 0.77 (0.64–0.92). However, subgroup analysis showed this benefit was lost in the 268 patients age ≥65 (47% of total trial population), with a HR of 0.96 (0.74–1.24) [31].

In 2011, the ACCORD-11 trial showed superiority of FOLFIRINOX regimen compared with Gemcitabine. In this phase III trial, 342 patients were randomized to the two treatment arms, and FOLFIRINOX was shown to have increased OS compared with Gemcitabine (11.1 months versus 6.8 months). However, the FOLFIRINOX arm had significantly more adverse side effects, including neutropenia, febrile neutropenia, and thrombocytopenia. Additionally, this trial only included patients with an ECOG score of ≤1, and excluded patients over age 75, with a median age of 61. The benefit, however, was seen in patients ≥65 with a HR of 0.48 (0.30–0.77), compared with a HR of 0.61 (0.46–0.82) in patients <65 [5].

In 2013, the MPACT trial showed that the addition of *nab*-Paclitaxel to Gemcitabine improved outcomes compared to Gemcitabine alone. In this trial, 861 patients were randomized into the two treatment arms, and the combination arm had greater overall survival (8.5 months versus 6.7 months) and better progression-free survival (6.4 months versus 3.3 months). This trial included patients from age 27–88, with a median age of 63. However, only patients with a Karnofsky performance status ≥70 were included. A subgroup analysis showed that the benefit on overall survival was not significant in those ≥65, with a HR of 0.81 (0.63–1.03) compared to a HR of 0.65 (0.53–0.79) in patients aged <65. However, the benefit on progression free survival was maintained in the older population, with a HR of 0.69 (0.52–0.91) compared with a HR of 0.69 (0.55–0.87) in those aged <65 [6].

These trials have brought us to our current standards of care for metastatic disease, which consists of FOLFIRINOX or nab-Paclitaxel with Gemcitabine, with less toxic agents used for patients with more comorbidities or poorer performance status. However, these trials focused much more on younger, fitter patients, therefore their generalizability to elderly and those with more comorbidities is unknown. Table 2 summarizes the articles that were selected for review of the management of metastatic disease.

### 3.2. How Do Elderly Fare with Treatment for Metastatic Disease Compared with Younger Patients?

We conducted a literature search to evaluate the data regarding outcomes of older adults receiving treatment for metastatic pancreatic cancer compared with younger patients. A retrospective study done by van der Geest et al. in the Netherlands looked at the Netherlands Cancer Registry and identified 9407 patients who had been diagnosed with metastatic pancreatic cancer between 2005 and 2013. They stratified this population by age and whether or not the patients received treatment, looking specifically at median overall survival. They found that elderly patients in both the treatment and untreated groups had significantly lower median survival times, with a median OS for ages <70, 70–74, 75–79, and >80 of 26, 27, 20, and 16 weeks. Survival times for untreated were 12,11,11, and 10 weeks respectively according to age group [32]. This study did not stratify treatment groups based on the type of treatment they received, so it is not surprising to see that elderly patients did worse considering they often have more comorbidities and are more likely to receive a reduced dose or less toxic and thereby less efficacious, form of chemotherapy.

### 3.3. How Do They Do with the Current Standard Therapies?

A study out of the University of Tokyo showed that, with gemcitabine therapy in metastatic disease, comorbidity, but not age, was a prognostic factor in predicting overall survival. This study analyzed 237 patients, 69 of whom were over the age of 75, and found that compared with patients with a Charleston comorbidity index (CCI) of 0, patients with a CCI of 1 and ≥2 had a hazard ratio for survival of 1.25 (95% CI 1.03–1.52) and 1.55 (95% CI 1.05–2.30), respectively. When stratified by CCI, elderly and young patients had similar overall survival advantage [33].

These results seemed to be supported by other, similar studies looking at gemcitabine-based regimens and survival in the elderly. A study by Marechal et al. looked at OS and TTP in 99 patients at their institution. They found no significant difference in OS (240 days versus 220 days) or TTP (119 days vs. 104 days) in those age <70 compared with patients over 70 [34].

Studies looking at FOLFIRINOX use in the elderly have yielded similar results. A retrospective study by Baldini et al. looked at 42 patients aged >70 who received FOLFIRINOX and found that they had a median OS of 11.6 months with a 12-month survival rate of 52.6%, similar to data from the ACCORD trial. All patients included in this analysis had a performance status ranging from 0–2. It was noted that toxicities reported in the elderly were actually less than that of the ACCORD trial, with only 23% reporting grade 3–4 toxicities, mostly neuropathy. However, a higher percentage of the elderly patients in this study required primary dose reductions (66%) compared with the ACCORD trial, suggesting a readiness to modify dosing according to anticipated toxicities [35]. 

Another retrospective study looking at FOLFIRINOX use in elderly by Berger et al. looked at 88 patients with metastatic disease who had received FOLFIRINOX and found that OS in patients age <65 was 11.2 months compared with those ≥65 where OS was 7.9 months, however this was not found to be a statistically significant difference (*p* = 0.83) [36].

### 3.4. What about Less Toxic Regimens, Such as FOLFIRI or FOLFOX?

A recent meta-analysis by Sonbol et al. demonstrated that FOLFIRI, but not FOLFOX demonstrated a survival advantage over single agent fluoropyrimidine (FP) therapy, as a second line treatment after progression on gemcitabine-based therapy. This analysis included 5 studies included 895 patients with a median age ranging between 61 and 65 for the 5 included manuscripts. The meta-analysis showed that adding irinotecan to FP therapy added an OS benefit (HR = 0.70; 95% CI 0.55–0.89) when compared to FP therapy alone. However, adding oxaliplatin to FP therapy did not show an OS benefit (HR = 1.03, 95% CI 0.64–1.67) [37].

While this review of studies that included older adults showed a benefit for FOLFIRI as second line therapy, there is limited to no data showing how it might perform as a first line therapy. Additionally, the results of this analysis were not specifically stratified by age groups. However, given the reduced toxicities of FOLFIRI and FOLFOX in comparison with FOLFIRINOX, this may be a reasonable option in older adults with poorer performance status, and more studies are needed to assess this potential therapy.

## 4. Conclusions

Our literature review finds that healthy and fit older patients should be offered the standards for ‘younger and fit’ patients though the survival benefit of surgery, radiation and chemotherapy may be diminished with advancing age. As always, a careful assessment of risks and benefits for each treatment modality need to be reviewed with the patient. This is especially true for surgical intervention, where the post-operative mortality increased with age (>70) and more than doubled in octogenarians who underwent surgical resection. 

Older patients had also been excluded from large randomized trials including ACCORD11 that excluded age >75 metastatic patients, and LAP-07 that excluded age >70 patients with locally advanced disease; therefore, limiting their generalizability to older patients. Interestingly, a subgroup analysis of the MPACT trial showed that the survival advantage of more intensive chemotherapy in metastatic patients age >65 was not statistically significant suggesting the need to examine the potential benefit of intensive chemotherapy in the older age groups. In addition, the standards-changing pivotal randomized trials had focused on ‘good performing’ patients and provided little to guide the treatment of those who are considered ‘treatable’ but with ‘borderline’ performance status. 

We advocate that more systematic studies should be conducted to evaluate the risk–benefit considerations of contemporary pancreatic cancer therapy in older patients, particularly those older than 75. The available evidence suggests that patients who are not candidates for aggressive therapy may still achieve a survival benefit with less toxic regimens such as single agent gemcitabine, FOLFOX, or FOLFIRI. Ideally, future studies will include a geriatric assessment to determine the fitness of the patient, which will help guide the patient and clinician to make the most appropriate choice among the various treatment regimens for the individual older patient with pancreatic cancer.

## Figures and Tables

**Table 1:** Articles comparing different treatments for localized Pancreatic Cancer.

Year/Article	Study Design	Median Age (Years), Range	Patient Population	Intervention	Outcome ^1^	AEs/Quality of Life
2017 [19]	RCT, phase III	65 (37–81)	Localized disease, underwent resection, n = 730	Gemcitabine + capecitabine vs. gemcitabine alone	Median OS 28 months vs. 25.5 months	608 grade 3–4 toxicities in combo group, 481 in control
2013 [17]	RCT, phase III	62 (34–82)	Localized disease, underwent resection, n = 368	Gemcitabine vs. observation	OS 22.8 months vs. 20.2, disease free survival 13.4 months vs. 6.7	N/A
2016 [23]	RCT, phase III	63 (57–71)	Locally advanced disease, n = 449	1st Randomization-gemcitabine or gem + erlotinib 2nd randomization-same chemo vs. chemorad. (54 Gy + capecitabine)	Median OS 16.5 months for chemo alone vs. 15.2 months for chemoradiotherapy	Similar b/t two groups
2018 [26]	Retrospective	Not given	Underwent resection, n = 727 (n ≥ 65 = 273)	Surgical resection young vs. elderly	Grade IIIB/IV post-op complications higher in older (16.8% vs. 9%, *p* = 0.002)	Overall complication rate 39.6% vs. 33% favoring younger
2013 [10]	Retrospective	65 (25–87)	Localized, underwent resection, n = 932 (<70 n = 580; 70–79 n = 288; >80 n = 64)	Surgical resection young vs. elderly	HR of 1.19 (0.85–1.66) for age 70–79 vs. <70, HR of 1.34 (0.75–2.38) for age ≥80	Not assessed
2016 [9]	Retrospective	67 (19–90)	Localized, underwent resection n = 3845	Surgical resection young vs. elderly	Elderly had higher 30-day mortality (4–5–7–8% for ages < 70, 70–74, 75–79, and >80, respectively *p* < 0.001), but similar 90-day and 3-year survival rates (6–10–13–12% 90-day mortality *p* < 0.001 and 35–33–28–31% 5-year survival *p* < 0.001)	Not assessed
2016 [7]	Retrospective	All patients age >65, median age not provided	Potentially resectable pancreatic AC, n = 2229	Surgical resection +/− chemo vs. chemotherapy alone	Longer OS for surgery group, attenuated with increasing age (15 months vs. 10 months overall, 13 vs. 10 in >80)	Not assessed
2017 [27]	Retrospective	All patients age >66, median age not provided	Localized, underwent resection, n = 4105	Surgical resection alone vs. surgery w/adjuvant chemo early (<12) and late (>12 months)	Early and late chemotherapy had better 6 months and 1 year survival vs. surgery alone with better outcomes in the late chemotherapy group (OR for early = 0.44 (0.35–0.53) and 0.71 (0.60–0.85) for 6 months and 1 year, respectively, late OR = 0.14 (0.10–0.17) and 0.51 (0.43–0.61)	Not assessed
2011 [8]	Retrospective	All patients age >66, median age not provided	Locoregional pancreatic CA diagnosis, n = 9553	Surgical resection	Age independent predictor of resection regardless of comorbidities compared to age < 70 (70–74 21% less likely, 75–79 = 47%, 80–84 = 72%, >85 94%), benefit of resection did not decrease with increasing age (16.1, 15.8, 14.9, 12.4, 12.3 mos. survival *p* = 0.08)	Not assessed
2017 [11]	Retrospective	(38–88) median age not provided	Underwent resection for localized PA, n = 227	Resection +/− adjuvant chemo	Median DFS of 15 months, 11 months, and 7 months for young (<70), elderly (70–80), and very elderly (>80), better for young (*p* = 0.012 and 0.016), median OS of 30 months, 20 months, and 14 months *p* = 0.07 and *p* < 0.001	Not assessed
2016 [12]	Retrospective	(18–90) median age not provided	Underwent resection for localized PA, n = 929	Resection	Similar 90 day mortality (3.2% vs. 5% in the younger vs. older, *p* = 0.09)	Not assessed
2016 [13]	Retrospective	68 (40–86)	Localized disease, underwent resection n = 206	Resection	Median OS was similar for young vs. old (23 and 17 months, *p* = 0.40), OS at 1, 3, and 5 years was 62%, 42%, and 25% in young, 56%, 28%, and 28% in old	No difference in complication rate (26% vs. 20% *p* = 0.41)
2016 [14]	Retrospective	<75 years n = 241 (44.9–74.9) median = 66 ≥ 75 n = 59 (75–88) median = 78	Underwent resection for localized PA, n = 300	Resection	Similar median OS for age <75 and ≥75 (19.2 vs. 18.4 months *p* = 0.175)	Not assessed
2015 [15]	Retrospective	<80 years n = 362 (mean = 64.7) >80 yrs n = 23 (mean = 82.6)	Underwent resection for localized PA, n = 385	Resection	Similar median OS for age <80 and >80 (21 vs. 19 months *p* = 0.86)	Not assessed
2015 [28]	Retrospective	82 (80–88)	Underwent resection for localized PA, n = 26	Resection +/− adjuvant chemo	Similar between those who received chemo vs. did not (1 year survival of 50% and 45%, MST of 12.4 and 11.7 months *p* = 0.263)	Not assessed
2014 [29]	Retrospective	<80 years n = 4102 (median = 65) ≥80 years n = 475 (median = 82)	Any age, underwent resection, n = 4577	Resection +/− adjuvant chemo	Age >80 had 2-fold increase in 30 day mortality than younger (OR = 2.0, 95% CI 1.3–3.1 *p* = 0.03)), similar to age 70–79 (OR 1.5, 95% CI 0.9–2.4 *p* = 0.120)	Not assessed

^1^ OS: Overall Survival; HR: Hazard Ratio; DFS: Disease Free Survival; OR: Odds Ratio; CI: Confidence Interval.

**Table 2:** Articles comparing treatment for metastatic disease.

Article/Year	Study Design	Median Age (Range)	Patient Population	Intervention	Outcome ^1^	AEs/Quality of Life
2011 [5]	Randomized trial, phase 3, multicenter, open-label	61 (25–76)	Patients with ECOG score of 0 or 1 with metastatic pancreatic CA, n = 342	FOLFIRINOX vs. Gemcitabine	Better OS in FOLFIRINOX (11.1 months vs. 6.8 months *p* < 0.001), better PFS (6.4 months vs. 3.3 months *p* < 0.001)	More adverse events in FOLFIRINOX (5.4% vs. 1.2% had febrile neutropenia, 9.1% vs. 3.6% thrombocytopenia, 45.7 vs. 21% neutropenia)
2013 [6]	Randomized trial, phase 3, multicenter, open-label	63 (27–88)	Advanced pancreatic CA w/ Karnofsky performance-status of 70 or more, n = 861	Gemcitabine alone vs. Gemcitabine + nab-paclitaxel	Better OS in combination group (8.5 months vs. 6.7 months, *p* < 0.001), better PFS (5.5 months vs. 3.7 months)	More grade 3 or higher AEs in combination group-neutropenia (38% vs. 27%), fatigue (17 vs. 7%) and neuropathy (17 vs. 1%)
2007 [31]	Randomized trial, phase 3, multicenter, open-label	63.9 (36.1–92.4)	pts with histologic or cytologic evidence of metastatic pancreatic AC with ECOG of <2, n = 569	Gemcitabine + Erlotinib vs. Gemcitabine alone	Better OS in combo group (6.24 months vs. 5.91 months) HR of 0.82 (0.69–0.99) *p* = 0.38 w/1-year survival rates of 23% and 17% (*p* = 0.023), PFS better in combo (3.75 months vs. 3.55 months, HR of 0.77 (0.64–0.92) *p* = 0.004)	Combination group had higher frequency of grade I/II A.E.s including rash, diarrhea, infection, stomatitis, no difference in grade III/IV
1997 [30]	Randomized trial, phase 3	62 (36–79)	Advanced symptomatic pancreatic cancer, n = 126	Gemcitabine vs. 5-FU	Clinical benefit in 23.8% of gemcitabine-treated patients vs. 4.8% of 5-FU (*p* = 0.0022), median OS 5.65 months vs. 4.41 months (*p* = 0.0025) 18% vs. 2% 1 year survival	No difference in AEs b/t groups
2016 [35]	Retrospective	73 (70–79)	Received FOLFIRINOX for advanced pancreatic AC, n = 42	FOLFIRINOX	Median OS was 11.6 months (95% CI 1–74), 12-month survival rate of 52.6% (95% CI 13.5–85.5), similar to ACCORD-11 trial	12 patients (29%) had grade 3/4 toxicity
2017 [36]	Retrospective	56 (32–78)	Histology proven pancreatic AC, irresectable, ECOG < 1, received FOLFIRINOX, n = 88	FOLFIRINOX	Median OS was not significantly different (11.2 months CI 8.9–13.6 for age <65, 7.9 months CI 5.8–10 for age >65, *p* = 0.83)	No significant difference in grade > 3 tox, 56.2% age < 65, 33.3% age > 65)
2016 [38]	Subgroup analysis	<70 n = 573; ≥70 n = 261 median/range not provided	Treated for unresectable pancreatic CA, n = 261	Gemcitabine + S-1, S-1 alone, or Gemcitabine alone	No significant diff in OS (10.2 GS, 8.0 S-1, 8.5 gemc), no difference in objective response rate (27.6%, 25.3%, 14.3%)	Grade >3 toxicities more frequent in GS group than S-1 or gem groups (*p* < 0.001 and *p* = 0.016, respectively)
2017 [39]	Retrospective	<65 n = 236 ≥65 n = 659 median/range not provided	Unresectable pancreatic CA, n = 895	Any chemotherapy vs. best supportive care (BCS)	Survival in chemotherapy group was similar by age (333 days for <65, 274 days for >65 *p* = 0.09) and similar in BSC (78 days vs. 84 days, *p* = 0.83)	Not assessed
2008 [34]	Retrospective	<70 n = 57 ≥70 n = 42 median/range not provided	Unresectable or metastatic pancreatic AC receiving gemcitabine-based chemo, n = 99	Gemcitabine-based chemotherapy	No difference in OS (240 days in <70 vs. 220 days in >70, *p* = 0.882) or TTP among elderly vs. young (119 days vs. 104 days, *p* = 0.846)	Similar rates between elderly and younger
2017 [32]	Retrospective	<70 n = 4729 70–74 n = 1623 75–79 n = 1437 ≥80 n = 1618 median/range not provided	Metastatic pancreatic CA diagnosis in the NCR, n = 9407	Any form of treatment or no treatment	Elderly had lower OS in treated (26 weeks, 27, 20, 16 for age < 70, 70–74,75–79, and >80 *p* = 0.003) and untreated (12,11,11,10 *p* < 0.001), administration of chemo increased b/t 2005-13 (26–43%, 14–25%, 5–13% (all *p* < 0.001), and 2–3% (not SS) for ages <70, 70–74, 75–79, and >80)	Not assessed
2014 [40]	Retrospective	73 (70–79)	Patients who started palliative 1st line chemo for advanced pancreatic CA, n = 53	First or second line palliative systemic chemotherapy	Elderly have similar OS and PFS rates to younger from trials (median PFS of 118 days in >75, median OS of 201 days (145.5 days for >75, 218 days for <75, *p* = 0.51)	30.2% experienced grade >3 tox, no significant difference between ECOG 0-1 vs. >2)
2011 [41]	Retrospective	75–84 (78)	Patients w/advanced or metastatic pancreatic AC, n = 38	Single agent gemcitabine according to the Burris regimen or GemOx regimen	Median OS was 8.9 months, similar to younger	23% experienced grade 3 toxicity (neutropenia), no grade 4, similar to younger
2010 [33]	Retrospective	67.8 +/− 10.7 (Mean +/− SD) <75 n = 168 ≥75 n = 69	Patients diagnosed with unresectable pancreatic CA, n = 237	Any treatment modality	Compared with CCI score of 0, CCI of 1 and >2 had a HR of 1.25 and 1.55, respectively. Age not a poor prognostic factor (OS between non elderly and elderly who received chemo was 10.8 and 10.9 months, respectively)	No difference in elderly vs. younger

^1^ OS: Overall Survival; HR: Hazard Ratio; DFS: Disease Free Survival; OR: Odds Ratio; CI: Confidence Interval; CCI: Charleston Comorbidity Index.
